# *5G2* mutant mice model loss of a commonly deleted segment of chromosome 7q22 in myeloid malignancies

**DOI:** 10.1038/s41375-024-02205-x

**Published:** 2024-03-05

**Authors:** Jasmine C. Wong, Kelley M. Weinfurtner, Tamara Westover, Jangkyung Kim, Eric J. Lebish, Maria del pilar Alzamora, Benjamin J. Huang, Michael Walsh, Sherif Abdelhamed, Jing Ma, Jeffery M. Klco, Kevin Shannon

**Affiliations:** 1 Department of Pediatrics, University of California, San Francisco, CA USA; 2 Department of Pathology, St. Jude Children’s Research Hospital, Memphis, TN USA; 3 Helen Diller Family Comprehensive Cancer Center, University of California, San Francisco, CA USA

**Keywords:** Cancer genetics, Myelodysplastic syndrome, Haematological cancer

## Abstract

Monosomy 7 and del(7q) are among the most common and poorly understood genetic alterations in myelodysplastic neoplasms and acute myeloid leukemia. Chromosome band 7q22 is a minimally deleted segment in myeloid malignancies with a del(7q). However, the rarity of “second hit” mutations supports the idea that del(7q22) represents a contiguous gene syndrome. We generated mice harboring a 1.5 Mb germline deletion of chromosome band 5G2 syntenic to human 7q22 that removes *Cux1* and 27 additional genes. Hematopoiesis is perturbed in *5G2*^*+/del*^ mice but they do not spontaneously develop hematologic disease. Whereas alkylator exposure modestly accelerated tumor development, the *5G2* deletion did not cooperate with *Kras*^*G12D*^, *Nras*^*G12D*^, or the MOL4070LTR retrovirus in leukemogenesis. *5G2*^*+/del*^ mice are a novel platform for interrogating the role of hemopoietic stem cell attrition/stress, cooperating mutations, genotoxins, and inflammation in myeloid malignancies characterized by monosomy 7/del(7q).

**TO THE EDITOR:**

Monosomy 7 (Mo7) and del(7q) [Mo7/del(7q22)] are highly prevalent chromosomal abnormalities in de novo pediatric and adult myelodysplastic neoplasm (MDS) and acute myeloid leukemia (AML) that are associated with an aggressive clinical course and therapeutic resistance [1, 2]. In addition, Mo7/del(7q) is highly enriched in myeloid malignancies that develop in patients with aplastic anemia or germline mutations in genes such as *NF1, SAMD9/9L*, and *GATA2* or following treatment with radiation or alkylating agents [3, 4]. In patients with Mo7/del(7q), the transformation from MDS to AML is characterized by recurring cooperating mutations in *NRAS/KRAS, SETBP1, RUNX1*, and other genes [5]. The lack of accurate in vitro and in vivo models is a major barrier to understanding how Mo7/del(7q) contributes to leukemogenesis.

Chromosome band 7q22 is a minimally deleted segment in MDS and AML samples that is syntenic with mouse chromosome band 5A3 [6] (Supplementary Fig. 1). Based on the rarity of “second hit” mutations in any 7q gene in myeloid malignancies with Mo7/del(7q), loss of 7q22 likely represents a contiguous gene syndrome whereby haploinsufficiency for multiple genes contributes to leukemogenesis [2]. Utilizing chromosome engineering, we previously generated *5A3*^*+/del*^ mice harboring a heterozygous germline deletion corresponding to part of this minimally deleted segment, which is bounded by *Fbxl13* and *Srpk2* [7] (Supplementary Fig. 1). The 5A3 deletion impairs lymphoid repopulation and perturbs the hematopoietic stem cell (HSC) compartment without enhancing repopulating potential or initiating hematologic disease [7]. Studies of MDS and AML patient samples have implicated a second region of 7q22 flanked by *EPO* and *UPK3BL* in leukemogenesis. This interval contains *CUX1* and 27 other genes and is syntenic to mouse chromosome band 5G2 (Supplementary Fig. 1). Here we report the generation and analysis of *5G2*^*+/del*^ mice harboring a heterozygous germline *Epo-Upk3bl* deletion (Supplementary Fig. 2).

The frequency of bone marrow (BM) c-kit^+^, lin^-^, Sca^+^ (KLS) cells is reduced in *5G2*^*+/del*^ mice compared to wild-type (WT) littermates, which is primarily due to a decrease in CD150^neg^ multi-potent progenitors (Figs. 1a, b). Further analysis of the KLS CD48^neg^ HSC population showed an increase in the myeloid-biased CD150^hi^ cells (Fig. 1c). Interestingly, 5-bromo-2’-deoxyuridine (BrdU) labeling revealed a significant reduction in the proportion of *5G2*^*+/del*^ KLS CD48^neg^ HSC in the S phase of the cell division cycle and an increase in the G0/G1 fraction (Fig. 1d). After backcrossing *5G2*^*+/del*^ mice to the C57BL/6 strain, we mixed WT or *5G2*^*+/del*^ BM cells (CD45.2) with WT CD45.1 competitors from congenic BoyJ mice at a 1:1 ratio and transplanted them into irradiated recipients. *5G2*^*+/del*^ cells showed a modest reduction in competitive fitness that did not achieve statistical significance (Fig. 1e). Consistent with RT-PCR analysis of individual 5G2 genes (Supplementary Fig. 2d), RNA sequencing revealed a ~50% reduction in the expression levels of genes within the deleted 5G2 interval (Fig. 1f) and showed that gene sets associated with interferon responses/inflammation were uniquely and significantly down-regulated in KLS, CD48^-^, CD150^neg^ cells from *5G2*^*+/del*^ mice in comparison to WT controls (Supplementary Fig. 3).

**Fig. 1:**
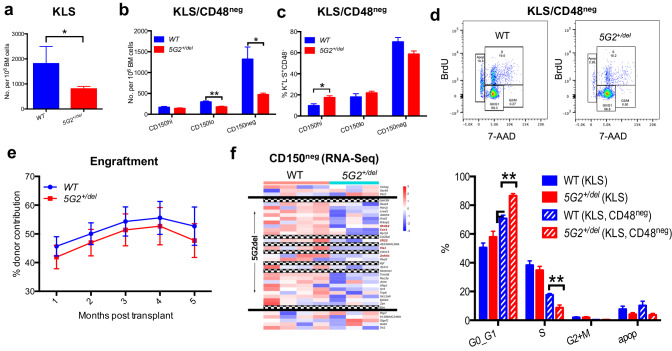
Characterization of *5G2*^*+/del*^ Mice. **a** Frequency of c-kit^+^, lin^-^, Sca^+^ (KLS) cells per 10^6^ nucleated BM cells in WT and *5G2*^*+/del*^ mice at 8–12 weeks of age (*n* = 5 per genotype). **b**, **c** Frequencies and percentages of CD150^hi^, CD150^lo^, and CD150^neg^ cells within the KLS, CD48^neg^ HSC population in WT and *5G2*^*+/del*^ mice. **d** BrdU staining showing the percentages of nucleated WT and *5G2*^*+/del*^ KLS, CD48^neg^ HSC in the G_0_/G_1_, and S + M phases of the cell cycle. **e** Percentage of blood leukocytes derived from WT and *5G2*^*+/del*^ BM cells after transplantation with WT competitors at a 1:1 ratio into irradiated WT recipients. These data were pooled from three independent experiments (biologic replicates) with at least three recipients (technical replicates) in each experimental group. The procedures used for BM harvesting and processing, flow cytometry, cell sorting, competitive repopulation, RNA isolation, and TaqMan analysis have been described in detail [7]. Data from these experiments are presented as means ± SEM and statistical significance was determined by performing two-tailed Student’s *t*-tests unless stated otherwise. Asterisks denote significant differences between WT and *5G2*^*+/del*^ mice (*p < 0.05; **p < 0.01). **f** Transcriptome profiling of KLS, CD48^neg^, CD150^neg^ HSCs from WT and *5G2*^*+/del*^ mice demonstrating reduced expression of genes in the G2 cytoband of mouse chromosome 5 ordered by location. Genes within the region that were filtered out of differential gene expression analysis due to low overall expression are represented by checkered bands. The bounds of the G2 region are denoted by black bands. The expression levels of the five genes highlighted in red font were verified by RT-PCR in the same KLS, CD48^neg^,CD150^neg^ HSC population (see Supplementary Fig. 2d).

*5G2*^*+/del*^ mice and their WT littermates remained well with normal hematologic parameters at >1 year of age (Supplementary Fig. 4). Likewise, thymus and spleen weights and BM cellularity were similar in *5G2*^*+/del*^ and WT mice at euthanasia (data not shown). Given the frequent occurrence of *NRAS* and *KRAS* mutations in myeloid malignancies with Mo7/del(7q), we generated cohorts of *Mx1-Cre; Kras*^*G12D/+*^ and *Mx1-Cre; Nras*^*G12D/+*^ mice either lacking or harboring the 5G2 deletion and induced Cre recombinase expression by injecting them with a single dose of polyI-polyC at weaning [8, 9]. As expected [8], *Mx1-Cre; Kras*^*G12D/+*^ mice developed a fully penetrant myeloproliferative disorder characterized by splenomegaly and leukocytosis that was similar in both *5G2* genotypes (Supplementary Figs. 5a–c). Additionally, the heterozygous 5G2 deletion did not modify survival or hematologic phenotypes in *Mx1-Cre; Nras*^*G12D/+*^ mice [9](Supplementary Fig. 5d). Furthermore, WT and *5G2*^*+/del*^ mice injected with the MOL4070LTR retrovirus [10] had similar survival and developed the same spectrum of hematologic malignancies (Supplementary Fig. 5e).

Mo7/del(7q) is strongly associated with therapy-induced MDS/AML following treatment with radiation and/or alkylating agents [4]. Accordingly, we injected *5G2*^*+/del*^ mice and WT littermates with the alkylating agent N-nitroso-N-ethylurea (ENU). This experiment revealed modest cooperativity between the 5G2 deletion and ENU exposure in tumorigenesis (Fig. 2a). To investigate if ENU treatment modulates competitive fitness, we mixed *5G2*^*+/del*^ or WT BM with WT competitor cells and transplanted them into irradiated syngeneic mice. Beginning five weeks after transplantation, these recipients received two doses of ENU or control vehicle separated by 7 days and were euthanized four weeks later to measure BM chimerism. The proportion of *5G2*^*+/del*^ cells was significantly reduced in recipient mice after ENU treatment in comparison to mice transplanted with WT competitors (Fig. 2b) and this difference was maintained over time (Supplementary Fig. 6). We next used s similar experimental design to assess the impact of the 5G2 deletion on the competitive fitness of HSC expressing *Nras*^*G12D*^ from the endogenous locus. Specifically, mice were transplanted with CD45.2 BM cells from *Mx1-Cre; Nras*^*G12D/+*^*;5G2*^*+/del*^ or control *Mx1-Cre; Nras*^*G12D/+*^ mice at a 1:1 ratio with WT CD45.1 competitors, and half of them were injected with ENU 5 and 6 weeks later (Fig. 2c). Recipient mice were monitored for 5 months after transplantation or until they became moribund and required euthanasia. In the absence of ENU treatment, the contribution of *Nras*^*G12D/+*^;*5G2*^*+/del*^ double mutant cells to the HSC and myeloid compartments was reduced in recipient mice compared to control *Nras*^*G12D/+*^ cells while lymphoid repopulation was similar (Fig. 2d). By contrast, most of the recipients transplanted with either *Nras*^*G12D/+*^;*5G2*^*+/del*^ or *Nras*^*G12D/+*^*WT* cells that received ENU died prematurely, primarily from lymphoid malignancies, which precluded assessing competitive fitness.

**Fig. 2:**
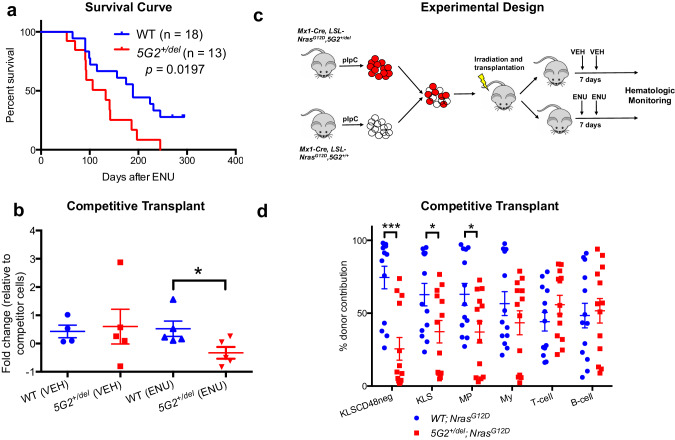
ENU Treatment of *5G2*^*del/+*^ Mice. **a** Survival of ENU-treated WT (*n* = 18) and *5G2*^*+/del*^ (*n* = 13) littermates. Percent survival (time to euthanasia of moribund animals) is plotted vs time in days after ENU treatment. The cumulative probability of survival was computed by the Kaplan-Meier method and is significantly less in *5G2*^*+/del*^ mice (*p* = 0.0197 by log rank). Thymic lymphoma was the major cause of death in mice of both genotypes. **b** Chimeras were generated by transplanting WT or *5G2*^*+/del*^ BM with WT competitor cells at a ratio of 3:1 into irradiated congenic WT recipients. Recipient mice received control vehicle or ENU at a dose of 100 mg/kg intraperitoneally 5 and 6 weeks post-transplant. The fold change in the percentage of total leukocytes derived from WT or *5G2*^*+/del*^ cells 4 weeks after the second ENU treatment is shown. Data represent the mean ± standard error of the mean (s.e.m.) of 4-5 mice. Chimerism was analyzed using Student’s *t*-test. **c** Experimental design for assessing the effects of the *5G2* deletion on the competitive fitness of *Nras*^*G12D*^ BM cells in the presence and absence of ENU treatment. Three-week-old *Mx1-Cre; Nras*^*G12D*^ and *Mx1-Cre; Nras*^*G12D*^*; 5G2*^*+/del*^ mice were injected with a single dose of pIpC to induce *Nras*^*G12D*^ expression from the endogenous locus followed by bone marrow harvest at 8-12 weeks for competitive transplantation with WT competitor cells at a 1:1 ratio. Recipient mice were treated with a control vehicle or ENU at 5 and 6 weeks post-transplant as above and were monitored for 5 months post-transplant. **d** Contribution of donor *Nras*^*G12D*^ (blue circles) and *Nras*^*G12D*^*; 5G2*^*+/del*^ (red squares) cells to HSC, KLS, myeloid progenitor (MP), Myeloid, T cell and B cell populations in the BM of recipient mice that did not receive ENU post-transplant. Note that the *5G2* mutation impaired the ability of donor *Nras*^*G12D*^ cells to repopulated the HSC, KLS, and MP compartments. Asterisks denote significant differences between *Nras*^*G12D*^ (blue circles) and *Nras*^*G12D*^*; 5G2*^*+/del*^ donor cells (*p < 0.05; ***p < 0.005 by Student’s *t*-test). Comparable competitive fitness data are not available from recipients that received ENU because most died prematurely from lymphoid malignancies.

Aly and colleagues identified 55 mutations and 6 microdeletions in the 7q22 gene *CUX1* in 1480 adults with MDS, MPN, or AML (4.1%) [11]. Of these mutations, 85% were heterozygous and 75% encoded missense amino acid substitutions. Patients with MDS had the highest incidence of *CUX1* mutations (~25%). Mo7/del(7q) was present in 81 additional cases (5.5%), including 5 with *CUX1* mutations [11]. By contrast, a comprehensive molecular analysis of 77 pediatric MDS and MDS/MPN patient samples revealed Mo7/del(7q) in 40% and *PTPN11, NRAS*, and other Ras pathway mutations in 55% [12]. Germline mutations in the 7q21.2 genes *SAMD9* and *SAMD9L* were identified in 17% of these pediatric cases and were strongly associated with loss of the chromosome 7 homolog harboring the mutant allele. The two *CUX1* mutations occurred in samples with concurrent Ras pathway mutations and Mo7/del(7q) [12]. These data and other studies indicate that monosomy 7 is more common than *CUX1* mutations in adult and pediatric myeloid malignancies, and demonstrate that *CUX1* is only rarely mutated in patients with monosomy 7 [2].

*Cux1* was independently investigated by two groups in mice using either an inducible knockdown approach [13, 14] or the *Vav-iCre* transgene to inactivate a conditional mutant allele [15]. In the first model, aged Cux1^Mid^ mice with a 45% reduction in Cux1 protein levels developed an indolent MDS-like disorder characterized by normal survival and blood leukocyte counts, an increase in the percentage of myeloid cells, anemia, dysplasia, and modest splenomegaly. HSC from these mice showed impaired self-renewal and *Cux1* knockdown cooperated with both ENU treatment and *Nras*^*G12D*^ expression in leukemogenesis [13, 14]. In the second model, heterozygous *Vav-iCre; Cux1*^*+/-*^ mice had normal blood leukocyte and erythrocyte numbers with macrocytosis at one year of age [15]. In agreement with these studies, we show that the heterozygous 5G2 deletion that removes *Cux1* neither alters lifespan nor causes acute leukemia. However, our findings in *Mx1-Cre; Kras*^*G12D/+*^ and *Mx1-Cre; Nras*^*G12D/+*^ mice haploinsufficient for the 5G2 deletion contrast with previous studies showing that *Flt3*^*ITD*^ or *Nras*^*G12D*^ cooperated with reduced *Cux1* expression in leukemogenesis [14, 15]. Haploinsufficiency for additional genes in the 5G2 interval - as commonly observed in human myeloid malignancies with Mo7/del(7q) - might account for these differences.

The HSC compartment of *5G2*^*+/del*^ mice is characterized by an increased percentage of CD150^hi^ myeloid-biased cells, delayed/reduced entry of HSC into the S phase of the cell cycle, and impaired competitive fitness after ENU treatment and in the context of *Nras*^*G12D*^ expression. The consistent absence of an in vivo growth advantage of *5A3*^*del/+*^*, 5G2*^*+/del*^, and *Cux1*^*Mid*^ HSCs under steady-state conditions is both intriguing and counter-intuitive as clonal outgrowth is a hallmark of myeloid malignancies. The discovery of germline *SAMD9* and *SAMD9L* mutations as a cause of familial MDS and AML suggests an alternative explanation for how Mo7/del(7q) might contribute to leukemogenesis [3]. HSC upregulate SAMD9 and SAMD9L as an adaptive response to inflammatory signals in the BM microenvironment. The *SAMD9* and *SAMD9L* mutations identified in pediatric MDS and AML encode biochemical gain-of-function proteins that inhibit Ras/mitogen-activated protein kinase (MAPK) signaling, suppress cell growth, perturb protein translation, and promote apoptosis, which favors the survival of Mo7/del(7q) clones that delete the mutant allele [3, 16, 17]. Knock-in mice harboring a conditional *Samd9l* mutant allele that models a mutation in familial MDS and AML develop BM aplasia with impaired HSC function that is exacerbated by inflammatory stress and associated with loss of the chromosomal segment harboring the mutant allele [18]. Our observation that gene sets associated with interferon signaling are down-regulated in *5G2*^*+/del*^ HSC provides a biologic rationale for why 7q22 deletions might be co-selected with loss of mutant *SAMD9/9L* alleles in response to inflammatory stress. Similarly, it is possible that haploinsufficiency for *SAMD9, SAMD9L*, and 7q22 cooperatively provide Mo7/del(7q) stem and progenitor cells with a survival – but not a proliferative - advantage in other disease settings characterized by chronic HSC stress/attrition [1, 19].

Together with a previous analysis of *5A3*^*+/del*^ mice [7], the studies of *5G2*^*+/del*^ mice reported here and observations in human patients suggest that Mo7/del(7q) functions as an “opportunistic” molecular abnormality in the context of HSC damage and dysfunction [1]. This idea is conceptually concordant with the outgrowth of *TP53* mutant clones in therapy-induced AML [20]. The prevalent mutations in *NRAS* and other signaling genes in myeloid disorders with monosomy 7/del(7q) may partially overcome the fitness disadvantage associated with *CUX1* haploinsufficiency and loss of the 7q22/5G2 interval by promoting cell cycle progression [14]. The distinct effects of the 5G2 and 5A3 deletions on HSC homeostasis is likely due to the fact that they independently removed 28 and 13 non-overlapping genes syntenic to different human 7q22 DNA segments. *5G2*^*+/del*^ mice are a genetically accurate model of the proposed 7q22 contiguous gene deletion syndrome for interrogating the role of Mo7/del(7q) in HSC homeostasis and for characterizing how a pro-inflammatory BM microenvironment shapes HSC survival, clonal evolution, and progression to MDS and AML. Future studies may also further elucidate the respective biologic and phenotypic consequences of haploinsufficiency for *Cux1* and other individual 5G2 genes versus loss of the entire *Upk3bl*-*Epo* interval.

### Supplementary information

Supplemental Material
