# Supplementary material for: 5G2 mutant mice model loss of a commonly deleted segment of chromosome 7q22 in myeloid malignancies
Source: Leukemia. 2024 Mar 5;38(5):1182–6. doi: 10.1038/s41375-024-02205-x (PMC11073961; doi:10.1038/s41375-024-02205-x)
Supplement: Supplementary file 1 — Supplemental Material [file 41375_2024_2205_MOESM1_ESM.pdf]

Supplementary Materials for "*5G2* Mice Model Loss of a Commonly Deleted Segment of Chromosome 7q22 in Myeloid Malignancies"

Supplementary Figure 1: Commonly-deleted 7q22 DNA segments and synteny to mouse chromosome bands *5A3* and *5G2.*

Supplementary Figure 2: Generation of $5G2^{+/del}$ mice.

Supplementary Figure 3: Pathway analysis of transcriptome data from $5G2^{+/del}$ HSCs.

Supplementary Figure 4. Hematologic parameters in wild-type (WT) and $5G2^{+/-}$ mice euthanized at age 64 weeks.

Supplementary Figure 5. Survival of $5G2^{+/del}$ and WT mice expressing $Kras^{G12D}$, $Nras^{G12D}$ or injected with the MOL4070LTR retrovirus.

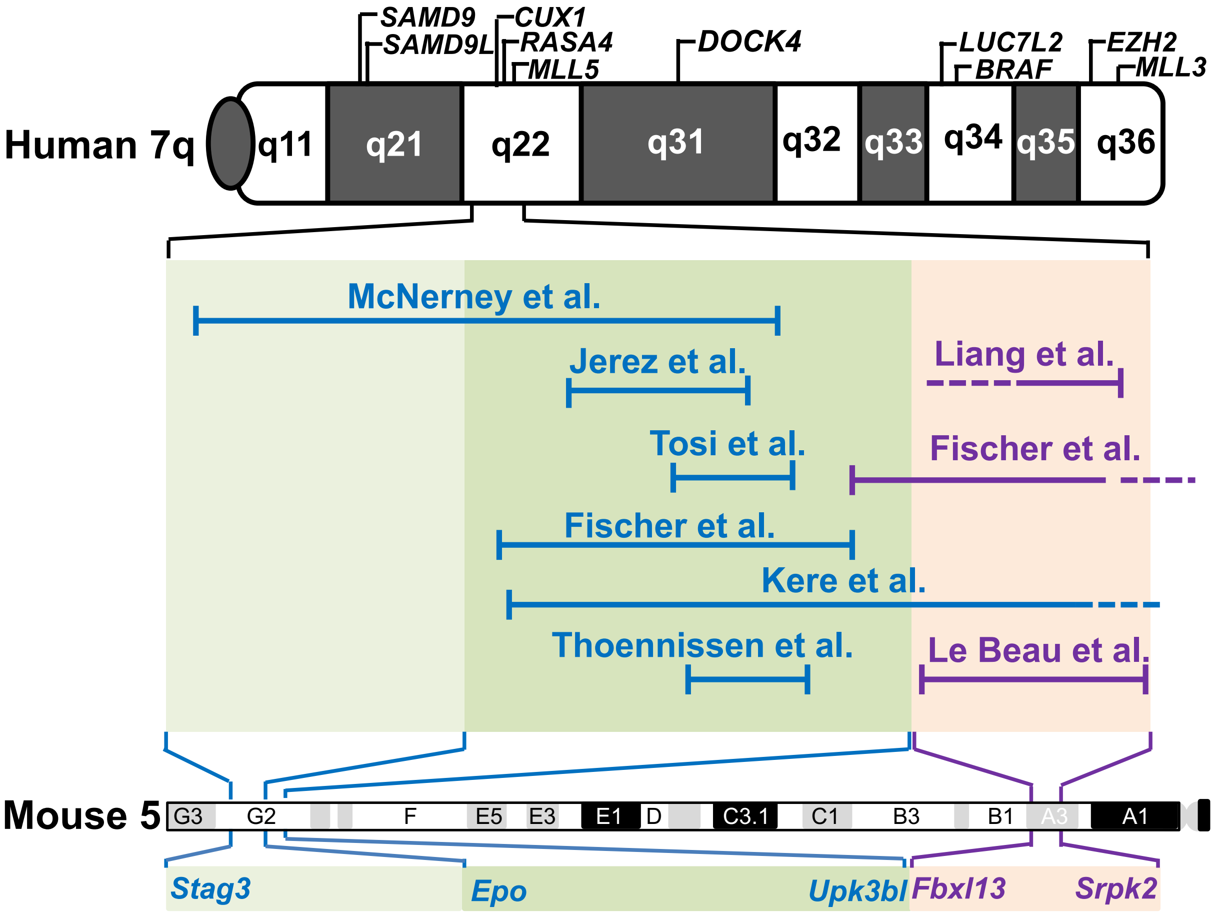

Supplementary Figure 1. Commonly-deleted 7q22 DNA segments and synteny to mouse chromosome bands *5A3* and *5G2*. The chromosomal locations of known and candidate 7q genes involved in leukemogenesis including *SAMD9, SAMD9L, CUX1, MLL5 (KMT2E), LUC7L2, EZH2,* and *MLL3 (KMT2C)* (1-7) are shown at the top. Commonly deleted 7q22 intervals defined by different research groups (3, 5, 8-13) appear in the center with tan and green boxes depicting segments that are syntenic to mouse chromosomes 5A3 and 5G2, respectively. A schematic showing mouse chromosome 5 and the boundary loci used to the create a previous *5A3*$^{+/del}$ *(Fbxl13-Srpk2)* strain(14) and the *Upk3bl-Epo* deletion of 5G2 presented in this report appear at the bottom. *SAMD9/9L* (human) and *Samd9l* (mouse) are outside the respective human commonly-deleted 7q22 and mouse 5A3 and 5G2 intervals. *Cux1* and *Rasa4* are within the 5G2 deletion reported here and *Mll5 (Kmt2e)* is in the 5A3-deleted interval (14).

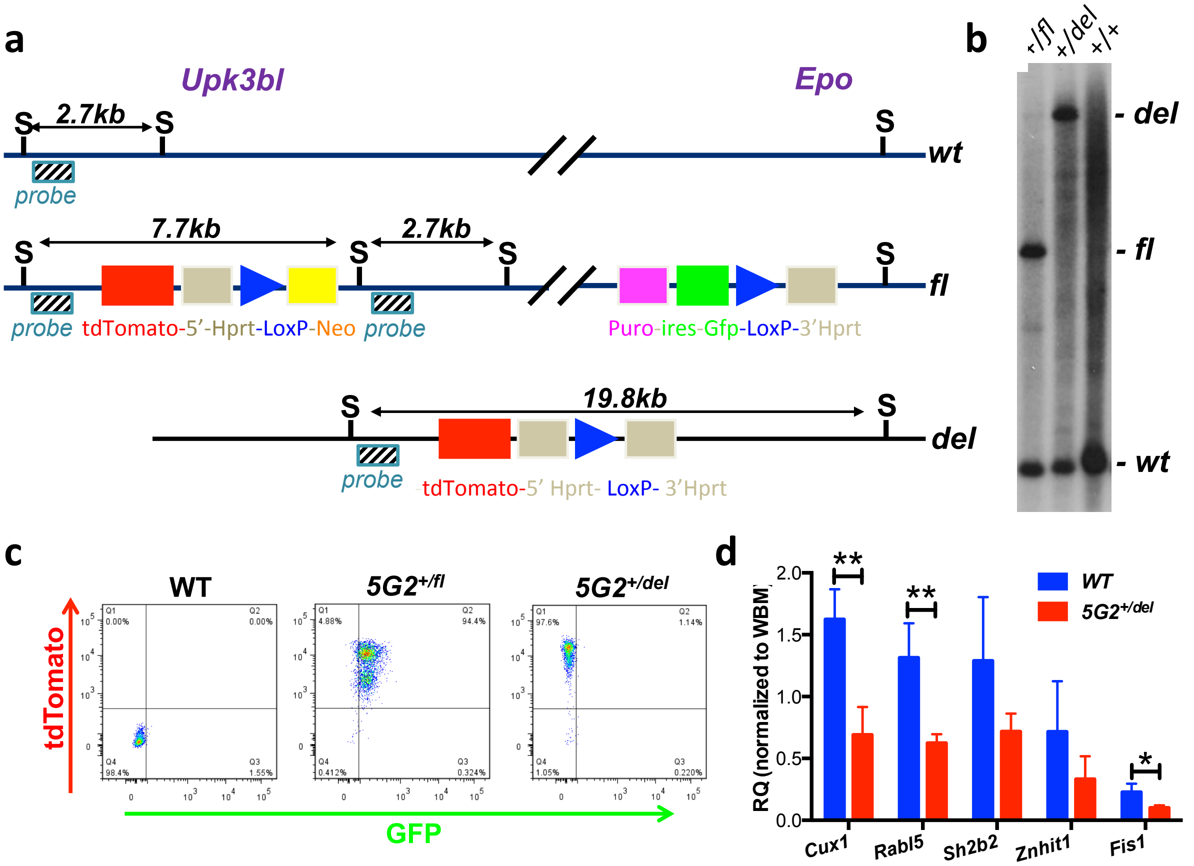

Supplementary Figure 2. *5G2$^{+/del}$* Mice harbor a *Upk3bl-Epo* deletion and show reduced expression of embedded genes. (**a**) Sequential gene targeting was performed at the flanking *Upk3bl* and *Epo* loci in mouse embryonic stem (ES) cells followed by Cre-mediated recombination to excise 1.5 Mb of genomic DNA. (**b**) Southern blot analysis demonstrating germline transmission of the latent mutant allele (+/fl) and the 5G2 deletion (+/del) by restriction digest of bone marrow DNA with StuI (S) enzyme. (**c**) tdTomato and green fluorescent protein (GFP) expression (middle panel) and tdTomato (right panel) expression in blood leukocytes from *5G2$^{+/fl}$* and *5G2$^{+/del}$* mice, respectively. (**d**) RT-PCR showing reduced expression of genes within the deleted *Upk3bl-Epo* interval in 5G2$^{+/del}$ KLS, CD150^{neg}, CD48^{neg} hematopoietic stem cells (HSC).

Detailed Methods. *5G2$^{+/del}$* mice harboring a tdTomato reporter at the *Upk3bl-Epo* deletion were generated by performing sequential rounds of gene targeting and clonal selection in mouse embryonic stem cells followed by blastocyst injections to generate chimeric mice. These general procedures have been reported (14). Study mice were housed in a specific pathogen–free facility at the University of California San Francisco, and all animal experiments were conducted under protocols approved by the Institutional Animal Care and Use Committee. Genotyping and disease monitoring were performed as previously described (14). Both 5' and 3' MICER backbone vectors were obtained from the Sanger Institute (UK). The 5' MICER backbone was modified by

adding a pCAGGs-tdTomato sequence into the *AvrII* and *HindII* sites with a Gateway reading frame A into the AscI site. A 8.9 kb fragment of genomic DNA located within the *Upk3bl* gene was PCR amplified from MICER Clone MHPP133j20 and inserted into a pENTR vector with the pENTR/D-TOPO kit. A Gateway LR reaction was performed between this entry vector and the modified 5' MICER backbone, resulting in the final targeting construct. E14 ES Cells (129P2 strain) were electroporated with *KpnI*-linearized targeting vector, and the clones were selected in G418. Correct gene targeting event was assessed by digesting ES cell DNA with *EcoRI* and hybridizing Southern blots with a 0.7 kb probe containing sequences downstream of the genomic DNA used to construct the targeting vector. Three positive clones were expanded for a second round of targeting with a new targeting vector containing the 3' MICER backbone. The 3'MICER backbone was modified to include GFP expression by the addition of an IRES-GFP to the PGK-Puro already present, using the *SacII* and *PacI* sites. A Gateway reading frame A was also added to the backbone to the *AscI* site. A 7.4 kb fragment of genomic DNA located upstream of the *Epo* gene was PCR amplified from MICER Clone MHPP30I19 and cloned into a pENTR vector using the pENTR/D-TOPO kit. A Gateway LR reaction was performed between this new pENTR vector and the modified 3' MICER backbone, resulting in the second targeting construct. Two singly targeted clones were electroporated with *AflII*-linearized targeting vector, and clones were selected with puromycin. The correct gene targeting event was assessed by digesting ES cell DNA with *StuI* and hybridizing Southern blots with a 0.3 kb probe containing sequences downstream of the genomic DNA used to construct the targeting vector.

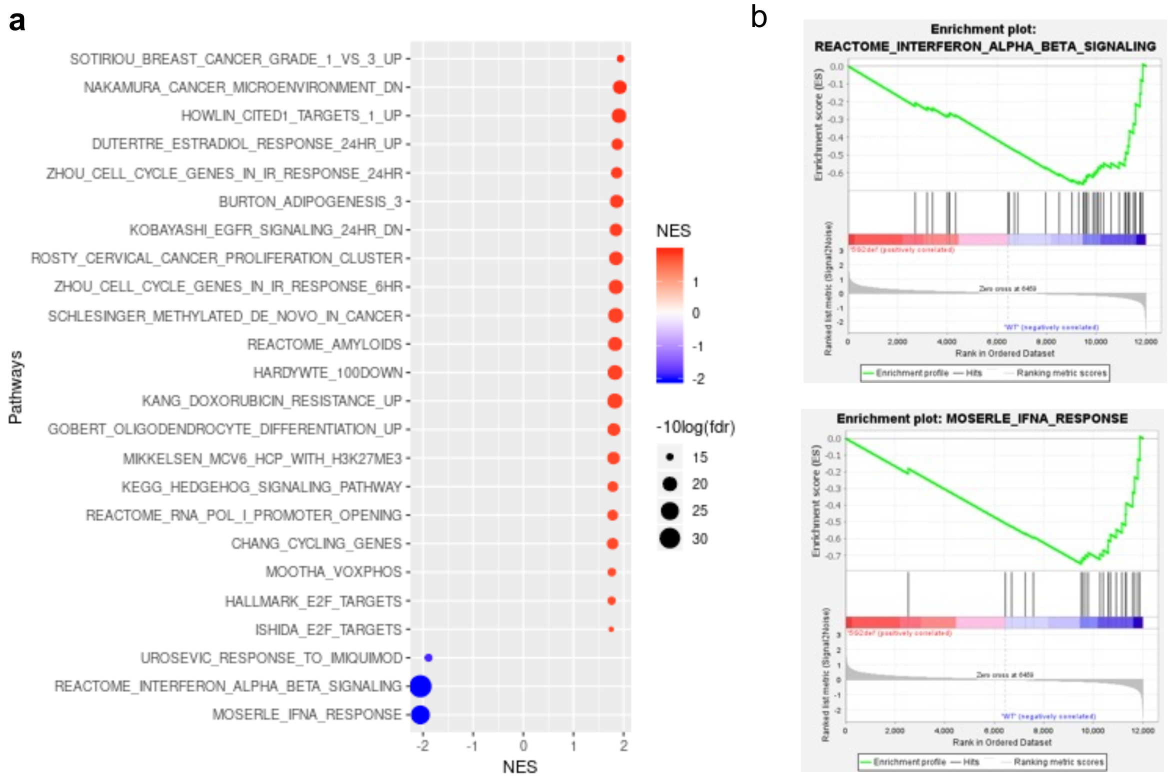

Supplementary Figure 3. Pathway analysis of HSCs from *5G2$^{+/del}$* mice. a. An enrichment plot of GSEA curated pathways enriched for genes differentially expressed in KLS, CD48^{-}, CD150^{neg} HSCs with deletions in the G2 band of mouse chromosome 5. A cutoff of fdr<.25 was used to determine which pathways were significantly enriched. **b.** Representative GSEA plots of downregulated pathways in A. RNA-seq analysis was performed by first isolating total RNA from CD150^{neg} HSCs (c-kit^{+}, lin^{-}, Sca^{+} (KLS), CD48^{-}) cells and converting it to double-stranded cDNA using the Tecan Ovation RNA sequencing system v2 (TECAN, CA). cDNA was sheared using the Covaris LE220 focused ultra sonicator (Covaris, MA) with a target size of 300 base pairs. Sheared cDNA was used to generate RNA-seq libraries using the KAPA Hyper-Prep kit (Roche, IN). Library quality and quantity were assessed by the Agilent DNA1000 Chip (Agilent, CA). Ten pM of each library was sequenced using Illumina SBS chemistry at 2 x 100 bp reads on the HiSeq2000 (Illumina®, CA). The RNA-Seq paired-end reads were mapped to the mouse mm10 genome using STAR (15) and quantified using RSEM (16). Differential gene expression analysis was performed as previously described (17).

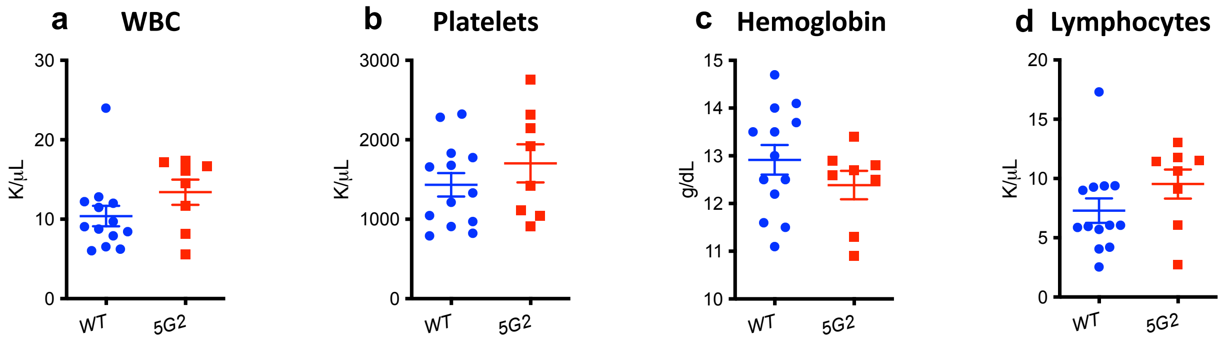

Supplementary Figure 4. Hematologic parameters in wild-type (WT) and *5G2$^{+/del}$* mice euthanized at age 64 weeks. (**a**) Blood leukocyte counts; (b) Hemoglobin values; (c) platelet counts; (d) lymphocyte counts.

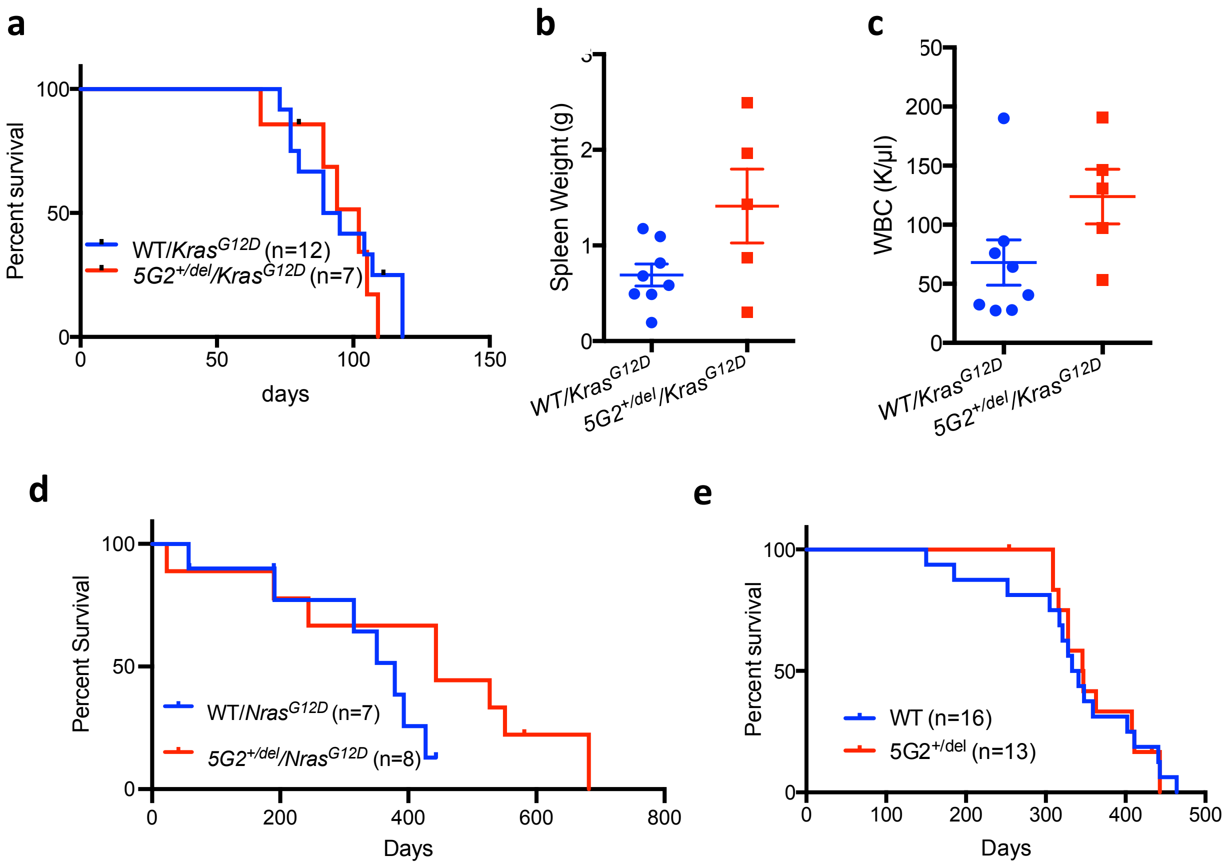

Supplementary Figure 5. Survival of *5G2*$^{+/del}$ and control mice expressing *Kras*^{G12D}/*Nras*^{G12D} or injected with the MOL4070LTR retrovirus. (**a**) Kaplan-Meier survival curve of control *Mx1-Cre, Kras*$^{LSL-G12D/+}$ *(WT/Kras*^{G12D}, n=12.) and *Mx1-Cre, Kras*$^{LSL-G12D}$*, 5G2*$^{+/del}$ littermates *(5G2*$^{+/del}$ */Kras*^{G12D}, n=7); p = 0.6294 by log rank. Percent survival (time to euthanasia of moribund animals) is plotted vs time in days. Spleen weights (**b**) and white blood cell (WBC) counts (**c**) of the mice shown in panel **a** at euthanasia. All mice died from progressive myeloproliferative disease as described previously *for Mx1-Cre, Kras*$^{LSL-G12D}$ mice (5). There were no statistically significant differences in spleen weights or WBC counts between the two groups and blasts were not present on blood smears at euthanasia. (**d**) Kaplan-Meier survival curve of control *Mx1-Cre, Nras*$^{LSL-G12D/+}$ *(WT/Nras*^{G12D}*,* n=7.) and *Mx1-Cre, Nras*$^{LSL-G12D/+}$*, 5G2*$^{+/del}$ littermates *(5G2*$^{+/del}$*/Nras*^{G12D}, n=8); p = 0.1658 by log rank. (**e**) Survival of *5G2*$^{+/del}$ mice and control littermates that were injected with the MOL4070LTR retrovirus as pups. Thymic lymphoma was the major cause of death in mice of both genotypes.

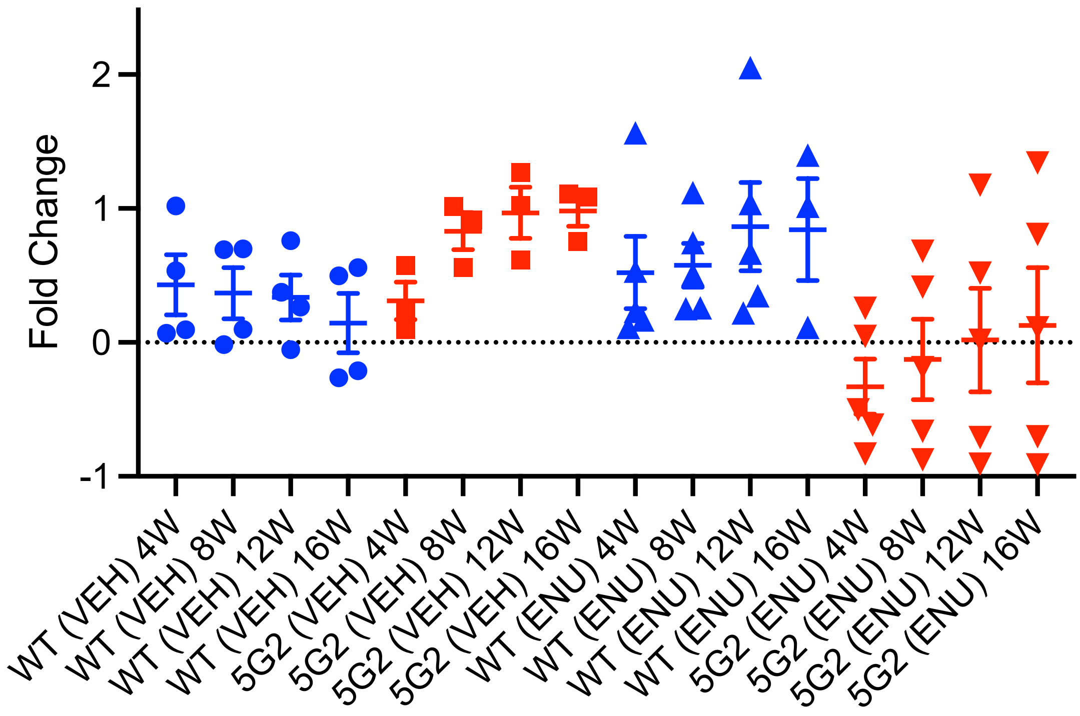

Supplementary Figure 6. Contribution of donor *Nras*^{G12D} (blue) and *Nras*^{G12D}*; 5G2*$^{+/del}$ (red) cells relative to WT competitor cells to blood chimerism 4-16 weeks after the second dose of ENU. Recipients of WT competitor cells are shown in blue with circles for control animals (no ENU treatment) and triangles for ENU-treated mice. Recipients of *5G2*$^{+/del}$ cells are displayed as red squares (no ENU) and triangles (ENU-treated), respectively. Figure 2c presents to overall design of this experiment.

Supplementary References

1. Chen C, Liu Y, Rappaport AR, Kitzing T, Schultz N, Zhao Z, Shroff AS, Dickins RA, Vakoc CR, Bradner JE, Stock W, LeBeau MM, Shannon KM, Kogan S, Zuber J, Lowe SW. MLL3 is a haploinsufficient 7q tumor suppressor in acute myeloid leukemia. Cancer Cell. 2014;25(5):652-65.
2. Ernst T, Chase AJ, Score J, Hidalgo-Curtis CE, Bryant C, Jones AV, Waghorn K, Zoi K, Ross FM, Reiter A, Hochhaus A, Drexler HG, Duncombe A, Cervantes F, Oscier D, Boultwood J, Grand FH, Cross NC. Inactivating mutations of the histone methyltransferase gene EZH2 in myeloid disorders. Nat Genet. 2010;42(8):722-6.
3. Hosono N, Makishima H, Jerez A, Yoshida K, Przychodzen B, McMahon S, Shiraishi Y, Chiba K, Tanaka H, Miyano S, Sanada M, Gomez-Segui I, Verma AK, McDevitt MA, Sekeres MA, Ogawa S, Maciejewski JP. Recurrent genetic defects on chromosome 7q in myeloid neoplasms. Leukemia. 2014;28(6):1348-51.
4. Nikoloski G, Langemeijer SM, Kuiper RP, Knops R, Massop M, Tonnissen ER, van der Heijden A, Scheele TN, Vandenberghe P, de Witte T, van der Reijden BA, Jansen JH. Somatic mutations of the histone methyltransferase gene EZH2 in myelodysplastic syndromes. Nat Genet. 2010;42(8):665-7.
5. McNerney ME, Brown CD, Wang X, Bartom ET, Karmakar S, Bandlamudi C, Yu S, Ko J, Sandall BP, Stricker T, Anastasi J, Grossman RL, Cunningham JM, Le Beau MM, White KP. CUX1 is a haploinsufficient tumor suppressor gene on chromosome 7 frequently inactivated in acute myeloid leukemia. Blood. 2013;121(6):975-83.
6. Papaemmanuil E, Gerstung M, Bullinger L, Gaidzik VI, Paschka P, Roberts ND, Potter NE, Heuser M, Thol F, Bolli N, Gundem G, Van Loo P, Martincorena I, Ganly P, Mudie L, McLaren S, O'Meara S, Raine K, Jones DR, Teague JW, Butler AP, Greaves MF, Ganser A, Dohner K, Schlenk RF, Dohner H, Campbell PJ. Genomic Classification and Prognosis in Acute Myeloid Leukemia. N Engl J Med. 2016;374(23):2209-21.
7. Thoennissen NH, Lasho T, Thoennissen GB, Ogawa S, Tefferi A, Koeffler HP. Novel CUX1 missense mutation in association with 7q- at leukemic transformation of MPN. Am J Hematol. 2011;86(8):703-5.
8. Le Beau MM, Espinosa R, 3rd, Davis EM, Eisenbart JD, Larson RA, Green ED. Cytogenetic and molecular delineation of a region of chromosome 7 commonly deleted in malignant myeloid diseases. Blood. 1996;88(6):1930-5.
9. Kere J, Ruutu T, Lahtinen R, de la Chapelle A. Molecular characterization of chromosome 7 long arm deletions in myeloid disorders. Blood. 1987;70:1349-53.
10. Fischer K, Frohling S, Scherer S, Brown J, Scholl C, Stilgenbauer S, Tsui L-C, Lichter P, Dohner H. Molecular cytogenetic delineation of deletions and translocations involving chromosome band 7q22 in myeloid leukemias. Blood. 1997;89:2035-41.
11. Tosi S, Scherer SW, Giudici G, Czepulkowski B, Biondi A, Kearney L. Delineation of multiple deleted regions in 7q in myeloid disorders. Genes Chromosomes Cancer. 1999;25(4):384-92.
12. Jerez A, Sugimoto Y, Makishima H, Verma A, Jankowska AM, Przychodzen B, Visconte V, Tiu RV, O'Keefe CL, Mohamedali AM, Kulasekararaj AG, Pellagatti A, McGraw K, Muramatsu H, Moliterno AR, Sekeres MA, McDevitt MA, Kojima S, List A, Boultwood J, Mufti GJ, Maciejewski JP. Loss of heterozygosity in 7q myeloid disorders: clinical associations and genomic pathogenesis. Blood. 2012;119(25):6109-17.

13. Liang H, Fairman J, Claxton DF, Nowell PC, Green ED, Nagarajan N. Molecular anatomy of chromosome 7q deletions in myeloid leukemia: Evidence for multiple critical loci. Proc Natl Acad Sci USA. 1998;95:3781-5.

14. Wong JC, Weinfurtner KM, Alzamora Mdel P, Kogan SC, Burgess MR, Zhang Y, Nakitandwe J, Ma J, Cheng J, Chen SC, Ho TT, Flach J, Reynaud D, Passegue E, Downing JR, Shannon K. Functional evidence implicating chromosome 7q22 haploinsufficiency in myelodysplastic syndrome pathogenesis. Elife. 2015;4.

15. Dobin A, Davis CA, Schlesinger F, Drenkow J, Zaleski C, Jha S, Batut P, Chaisson M, Gingeras TR. STAR: ultrafast universal RNA-seq aligner. Bioinformatics. 2013;29(1):15-21.

16. Li B, Dewey CN. RSEM: accurate transcript quantification from RNA-Seq data with or without a reference genome. BMC Bioinformatics. 2011;12:323.

17. Thomas ME, 3rd, Abdelhamed S, Hiltenbrand R, Schwartz JR, Sakurada SM, Walsh M, Song G, Ma J, Pruett-Miller SM, Klco JM. Pediatric MDS and bone marrow failure-associated germline mutations in SAMD9 and SAMD9L impair multiple pathways in primary hematopoietic cells. Leukemia. 2021;35(11):3232-44.
